# Comparison of Traditional Curettage to Endoscopic Techniques in Pediatric Adenoidectomy: A Systematic Review of Post-operative Outcomes

**DOI:** 10.7759/cureus.99188

**Published:** 2025-12-14

**Authors:** Asmaa AlShatti, Ahmad J Alali, Maha Al-Gilani

**Affiliations:** 1 Department of Otolaryngology - Head and Neck Surgery, Jaber Al-Ahmad Hospital, Kuwait City, KWT; 2 Department of Otorhinolaryngology, Kuwait Institute for Medical Specialization (KIMS), Kuwait City, KWT

**Keywords:** adenoidectomy, curettage, endoscopic coblation, endoscopic microdebrider, pediatric surgery

## Abstract

Adenoidectomy is a common pediatric procedure with generally low complication rates; however, postoperative ear pain, pressure symptoms, and Eustachian tube dysfunction remain reported concerns. This systematic review compares traditional curettage with endoscopic techniques - specifically endoscopic microdebrider adenoidectomy (EMA) and endoscopic coblation adenoidectomy (ECA) - in terms of postoperative outcomes in the pediatric population.

A systematic literature search was conducted across PubMed, Cochrane, and ScienceDirect databases between April and June 2024, following the Preferred Reporting Items for Systematic Reviews and Meta-Analyses (PRISMA) reporting guidelines. Studies published in English within the past 10 years were included. Only randomized controlled trials (RCTs) were eligible. Eleven RCTs met the inclusion criteria.

A total of 11 studies were included. Evaluated outcomes included middle ear pressure, hearing threshold, intraoperative time, bleeding, postoperative pain, and residual adenoid tissue. Endoscopic techniques demonstrated superior short-term postoperative results, with lower pain scores and improved middle ear pressure within the early recovery period; however, these differences were largely transient. Traditional curettage was associated with shorter operative time and slightly less intraoperative bleeding, while endoscopic techniques achieved more complete adenoid resection.

Endoscopic adenoidectomy - particularly coblation - offers improved short-term recovery and more complete tissue removal compared to traditional curettage, though early advantages tend to normalize over time. Further large-scale RCTs with standardized pain scoring and cost analysis are warranted to optimize surgical decision-making.

## Introduction and background

Adenoidectomy, the surgical removal of hypertrophic adenoid tissue, is among the most commonly performed procedures in pediatric otolaryngology. In the United States, approximately 250,000 adenoidectomies are performed annually [1]. According to the American Academy of Otolaryngology-Head and Neck Surgery, the procedure is indicated for patients with persistent or recurrent adenoiditis despite adequate medical therapy, recurrent otitis media, or sleep-disordered breathing due to nasopharyngeal obstruction [2].

Although generally regarded as safe, adenoidectomy carries a range of potential complications, varying from minor to major events requiring further intervention. Minor complications include otalgia, transient fever, sore throat, and mild dehydration resulting from reduced oral intake [3]. 

Intermediate complications may involve velopharyngeal insufficiency (VPI), characterized by hypernasal speech or nasal regurgitation. VPI is usually transient but may persist in children with underlying palatal abnormalities [3]. 

Major complications, though rare, include postoperative hemorrhage, reported to occur at an estimated rate of 0.49% in the literature [4]. More serious but uncommon events include atlantoaxial subluxation and nasopharyngeal stenosis [3,5]. Extremely rare complications such as internal carotid artery injury and cervical osteomyelitis have been described in isolated case reports [6,7].

Traditional curettage remains the most commonly utilized technique for adenoid removal; however, endoscopic approaches - such as microdebrider-assisted adenoidectomy and coblation-assisted adenoidectomy - have gained prominence, offering improved visualization and potentially fewer complications. This systematic review aims to synthesize current evidence comparing these surgical techniques, with a particular focus on the following postoperative outcomes: middle ear pressure, hearing threshold, intraoperative time, bleeding, pain, and residual adenoid tissue.

## Review

Methods 

This review protocol has been registered in the International Prospective Register of Systematic Reviews (PROSPERO) (ID CRD42024547443). Data base search was conducted between April and June 2024, the Preferred Reporting Items for Systematic Reviews and Meta-Analyses (PRISMA) reporting guidelines were followed [8]. An electronic search was done on different databases including PubMed, Cochrane and ScienceDirect. Inclusion criteria included studies published in English and studies published in the past 10 years. Observational, non-comparative studies and case reports were excluded; only randomized controlled trials (RCTs) were included. No geographic restrictions were applied during study selection; the geographic distribution of included studies reflects the availability of published literature during the search period.

The following search strings were used in all databases: “Adenoidectomy” OR “Adenoid resection” AND “Eustachian tube function” OR “Middle ear pressure”.

The PICO criteria for this review were as follows: Population: Pediatric population undergoing adenoidectomy without tonsillectomy; Intervention: Endoscopic Microdebrider or Endoscopic Coblation; Comparison: Traditional curettage; Outcome: Middle ear pressure, eustachian tube function, hearing threshold, post-operative pain, and residual adenoids. 

Data was sought for the predefined outcomes listed above; not all papers were compatible with the predefined outcomes, only mentioned results were included and no assumptions were made for any missing information. Studies included in this review were assessed for quality and bias using The Cochrane Collaboration’s tool for assessing risk of bias in randomized trials by a single reviewer (Table 1).

**Table 1:** Cochrane Collaboration’s tool for assessing risk of bias in randomized trials.

Study author (date)	Selection bias	Performance bias	Detection bias	Attrition bias	Reporting bias
Atilla et al. (2020) [9]	Unclear	High	Low	Unclear	Low
Singh et al. (2019) [15]	Low	Unclear	High	Unclear	Low
Juneja et al. (2019) [14]	Low​​​​​​​	High​​​​​​​	Unclear​​​​​​​	Unclear​​​​​​​	Low​​​​​​​
Gulsen et al. (2019) [12]	Low​​​​​​​	Moderate​​​​​​​	Unclear​​​​​​​	Unclear​​​​​​​	Low​​​​​​​
Kozcu et al. (2019) [10]	Low​​​​​​​	Moderate​​​​​​​	Unclear​​​​​​​	Unclear​​​​​​​	High​​​​​​​
Singh et al. (2020) [19]	Low​​​​​​​	Moderate​​​​​​​	Low​​​​​​​	Unclear​​​​​​​	Low​​​​​​​
Rajan et al. (2020) [11]	Low​​​​​​​	Moderate​​​​​​​	Low​​​​​​​	Unclear​​​​​​​	Low​​​​​​​
Abo Elmagd et al. (2021) [16]	Moderate	Moderate​​​​​​​	High​​​​​​​	Unclear​​​​​​​	Moderate​​​​​​​
Subash et al. (2022) [18]	Low​​​​​​​	Moderate​​​​​​​	High​​​​​​​	Unclear​​​​​​​	Unclear
Wadia et al. (2022) [17]	Low​​​​​​​	Moderate​​​​​​​	Unclear​​​​​​​	Unclear​​​​​​​	Unclear
Seleim et al. (2024) [13]	Moderate​​​​​​​	Low​​​​​​​	Unclear​​​​​​​	Unclear​​​​​​​	Unclear

**Figure 1:**
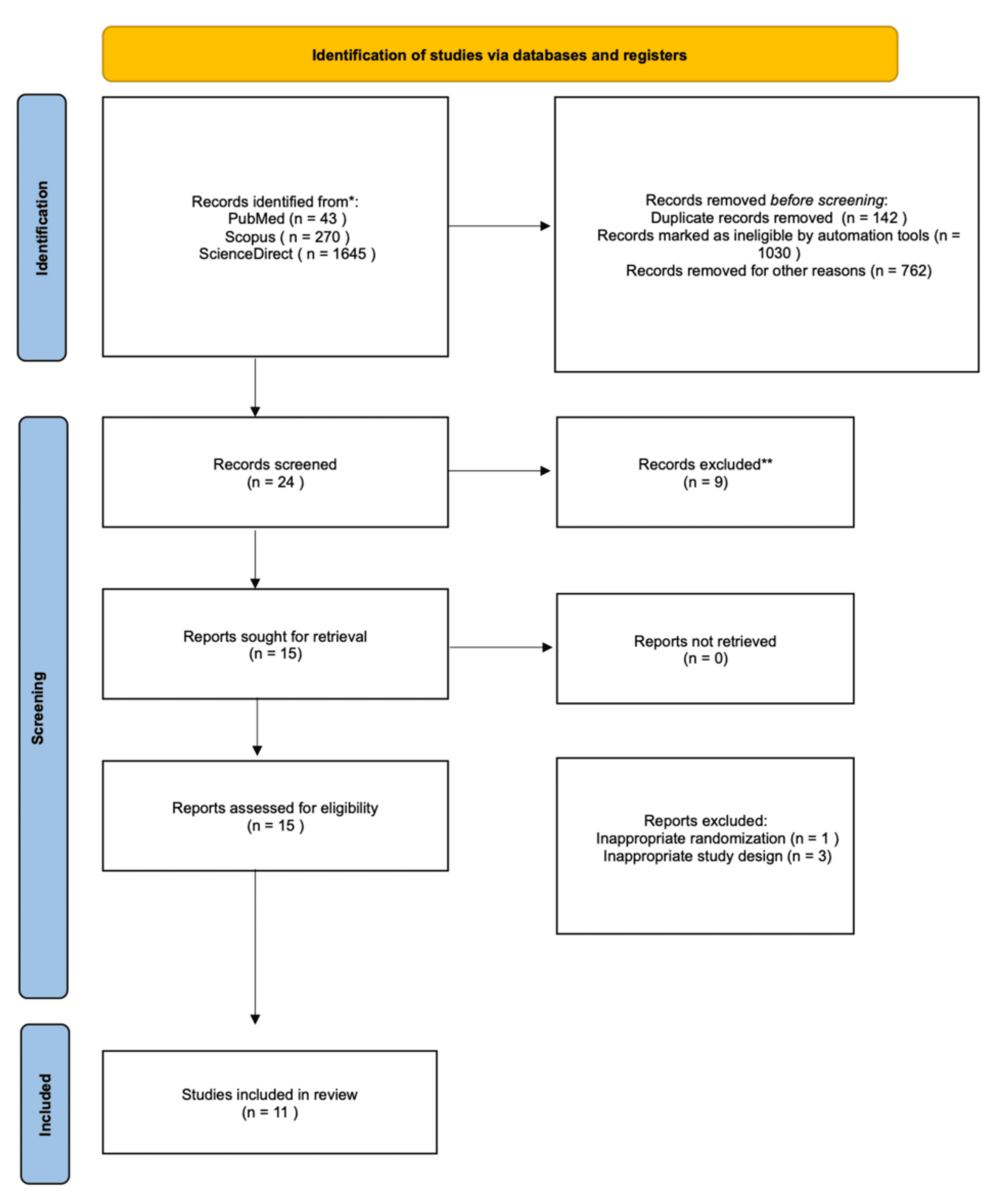
Preferred Reporting Items for Systematic Reviews and Meta-Analyses (PRISMA) chart illustrating identification and screening process.

Data extraction was done by two authors independently on a shared Excel sheet (Microsoft, Redmond, WA, USA). Disagreements were resolved by the project supervisor. Results were plotted on an Excel sheet and Excel was used to generate schematic figures when appropriate. In terms of heterogeneity, only papers discussing pediatric patients were included; subgroup analysis was done when indicated (e.g. different pain scores). Figure 1 is the PRISMA flowchart. Table 2 shows the characteristics of included studies. 

**Table 2:** Study characteristics. SD: standard deviation, RCT: randomised controlled trial, ECA: endoscopic coblation adenoidectomy, EMA: endoscopic microdebrider adenoidectomy

Author (Year)	Journal	Study Design	Population	Age (Mean ± SD)	Sex (M:F)	Total Sample	Intervention Group		Control Group
Atilla et al. (2020) [9]	Brazilian Journal of Otolaryngology	RCT	Pediatric patients with a normal tympanic membrane and normal tympanogram	Curettage = 90.43 ± 35.92, EMA = 83.15 ± 26.78 (Months)	Curettage 15:17, EMA 16:17	65	EMA = 33		Curettage = 32
Singh et al. (2019) [15]	Dubai Medical Journal	RCT	Pediatric patients aged 6–12	Curettage 9.30±2.200, EMA 8.23±2.373 (Years)	Curettage 22:8, EMA 25:5	60	EMA = 30		Curettage = 30
Juneja et al. (2019) [14]	The Journal of Laryngology & Otology	RCT	Patients aged 4–12 years	Curettage 7.95, EMA 8.48 (Years)	Curettage 17:8, EMA 18:7	50	EMA = 25		Curettage = 25
Gulsen et al. (2019) [12]	The Journal of Craniofacial Surgery	RCT	Pediatric patients undergoing primary adenoidectomy	Curettage 6.9±3.1, ECA 7.2 ±4.5 (Years)	Curettage 18:18, Coblation 18:18	72	ECA = 36		Curettage= 36
Kozcu et al. (2019) [10]	International Journal of Pediatric Otorhinolaryngology	RCT	Pediatric patients undergoing primary adenoidectomy, aged 4-17 years	Curettage 7,07 ± 2,95 , EMA 7,83 ± 3,16 (Years)	Curettage 15:15, EMA 15:15	60	EMA = 30		Curettage = 30
Singh et al. (2020) [19]	Indian Journal of Otolaryngology and Head & Neck Surgery	RCT	Children less than 15 years of age.	ECA 59.6 +- 37.4, EMA 54.9 +-36.4 (Months)	Coblation 41:39, EMA 47:33	160	ECA = 80		EMA = 80
Rajan et al. (2020) [11]	European Archives of Oto-Rhino-Laryngology and Head & Neck	RCT	Pediatric patients	EMA (median age= 10 years, IQR = 4) Curretage =(median age= 9 years, IQR =5)	Curretage 29:34, EMA 26:37	126	EMA = 63		Curretage = 63
Abo Elmagd et al. (2021) [16]	The Egyptian Journal of Otolaryngology	RCT	Pediatric patients scheduled for adenoidectomy	Curettage 7.43 ± 2.87, EMA 7.27 ± 2.36 (Years)	Gender ratio was nearly equal.	60	EMA = 30		Curettage = 30
Subash et al. (2022) [18]	Journal of Cardiovascular Disease Research	RCT	Children aged 3–10 years with symptoms of adenoid hypertrophy	Curettage 6.25±2.47, ECA 5.85 ±1.98 (Years)	Curettage 11:9, Coblation 10:10	40	ECA = 20		Curettage = 20
Wadia et al. (2022) [17]	Indian Journal of Otolaryngology and Head & Neck Surgery	RCT	Children aged 5-15, requiring adenoidectomy	Curettage 8.76 ± 3.45, EMA 8.91 ± 3.21 (Years)	Male predominance (55%) was noted in study participants.	60	EMA = 30		Curettage = 30
Seleim et al. (2024) [13]	European Archives of Oto-Rhino-Laryngology and Head & Neck	RCT	Children aged from 3 to 12 years old with symptomatic adenoid hypertrophy	Curettage = 9.03 ± 1.87, EMA = 8.99 ± 2.08, ECA= 8.99 ± 2.08 (Years)	Curettage = 18:12, EMA = 15:15, Coblation = 15:15	90	EMA= 30	ECA= 30	Curettage = 30

Results 

Tympanometry (Middle Ear Pressure) 

Middle ear pressure (tympanometry reading) post-operatively was one of the main primary outcomes of this paper. A total of five papers discussed differences in post-op tympanometry. Three papers discussed the difference between traditional curettage and endoscopic microdebrider adenoidectomy (EMA). Atilla et al. reported better middle ear pressure readings (daPA) in the EMA group on postoperative day (POD) 1 (p<0.001 for the right and left ear), however, there was no statistical significance between the two groups on POD7 (right p=0.338, left p=0.446) [9]. This is consistent with the findings of Kozcu et al., who also reported better middle ear pressure in both right and left ears in the EMA group on POD1 (p=0.046, p=0.049) respectively. On POD7, the EMA had superior middle ear pressure readings in the left ear (p= 0.025), but insignificant difference in the right ear [10]. On the contrary, Rajan et al. reported no statistical difference in mean improvement in middle ear function between the two groups at 12 weeks (p=0.40) [11]. 

One paper compared curettage to endoscopic coblation (ECA). Gulsen et al. reported better middle ear pressure readings in the ECA group in both right and left ear on POD1 (p<0.001 right and left), insignificant difference was reported on POD7 across the two modalities (right p=0.386, left p=0.517) [12]. 

Seleim et al. compared EMA, ECA and traditional curettage, although middle ear pressure readings were not reported, tympanogram types were reported, no statistical difference in tympanogram types was found between the three treatment modalities after two weeks and three months. However, after six months, the traditional curettage group was found superior to other modalities as 100% of this group had type A tympanogram compared to 85% in the EMA and 95% in the ECA (p<0.008) [13]. 

Overall, these studies indicate that early postoperative differences may exist favoring endoscopic techniques, but most follow-up assessments demonstrate no consistent long-term advantage, highlighting that both traditional and endoscopic approaches generally achieve comparable middle ear outcomes.

Hearing Threshold and Tympanic Membrane Compliance 

Rajan et al. tested 226 ears for improvement in hearing threshold, at 12 weeks, traditional curettage results were 2.54 ± 3.98 dB, compared to 3.20 ± 4.95 dB in the EMA group. There was no significance in hearing improvement between the two groups. There was no statistical significance between traditional curettage and EMA in terms of tympanic membrane compliance in mL at 12 weeks (p=0.07) [11]. 

Intra-operative Time 

**Figure 2:**
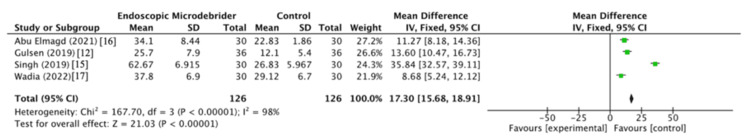
Traditional curettage versus endoscopic microdebrider adenoidectomy (EMA) in terms of intra-operative timing.

Seven papers compared intra-operative timing across the three surgical modalities. Juneja et al., Singh et al., Abo Elmagd et al. and Wadia et al. all concluded that traditional curettage procedure took a statistically significant shorter duration compared to EMA (Figure 2) [14-17]. 

Subash et al. and Gulsen et al. concluded that traditional curettage took statistically significant shorter duration than ECA [12,18]. Singh et al. (2020) reported an average of 22.08 minutes in the ECA group compared to an average of 12.78 minutes in the EMA group [19].

Intra-operative Bleeding

Five studies compared intra-operative bleeding between traditional curettage and EMA. Overall, there was a general trend for slightly lower blood loss with traditional curettage, although findings were inconsistent. Juneja et al. reported a mean blood loss of 46.8 mL for curettage versus 49.0 mL for EMA, which was not statistically significant (p>0.05) [14]. Singh et al. (2019) found a statistically significant reduction in blood loss with curettage (p<0.05) [15]. Abo Elmagd et al. also reported lower intra-operative bleeding with curettage, whereas Wadia et al. observed a similar trend that was not statistically significant (p=0.08) [16,17].

In comparisons with ECA, ECA was consistently associated with less intra-operative bleeding than traditional curettage. Gulsen et al. reported mean blood loss of 24.3 mL in the ECA group versus 43.8 mL in the curettage group (p<0.0001) [12], and Subash et al. reported similar findings (p=0.004) [18]. In a head-to-head comparison of EMA and ECA, Singh (2020) found that ECA resulted in significantly lower intra-operative bleeding than EMA (p<0.001) [19].

**Figure 3:**
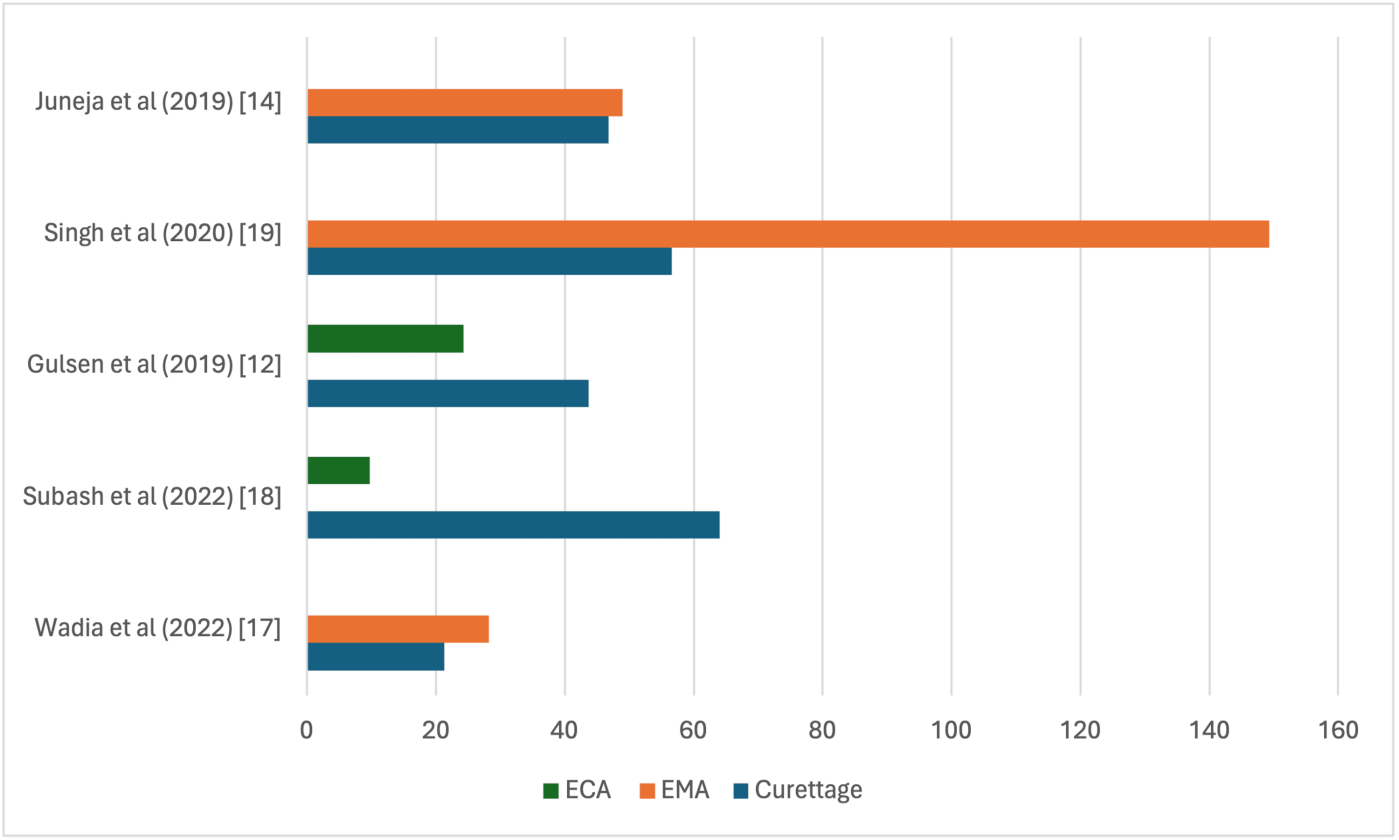
Mean intra-operative bleeding in mL, comparing the three surgical modalities. ECA: endoscopic coblation adenoidectomy, EMA: endoscopic microdebrider adenoidectomy

Overall, while traditional curettage generally resulted in slightly lower bleeding compared to EMA, and ECA tended to produce the lowest blood loss among the three techniques, the magnitude and statistical significance of differences varied across studies, suggesting that all three approaches are generally safe with respect to intra-operative bleeding (Figure 3).

Pain 

Ten papers discussed post-operative pain; however, different pain scores were used in these papers. Four papers reported pain using numerical pain scores (NPS), one papers used Wong Baker Faces (WBFs) score, two papers used Visual Analogue Scale (VAS) score and one paper used NPS and WBFs to report post-operative pain. 

EMA Versus Traditional Curettage 

**Figure 4:**
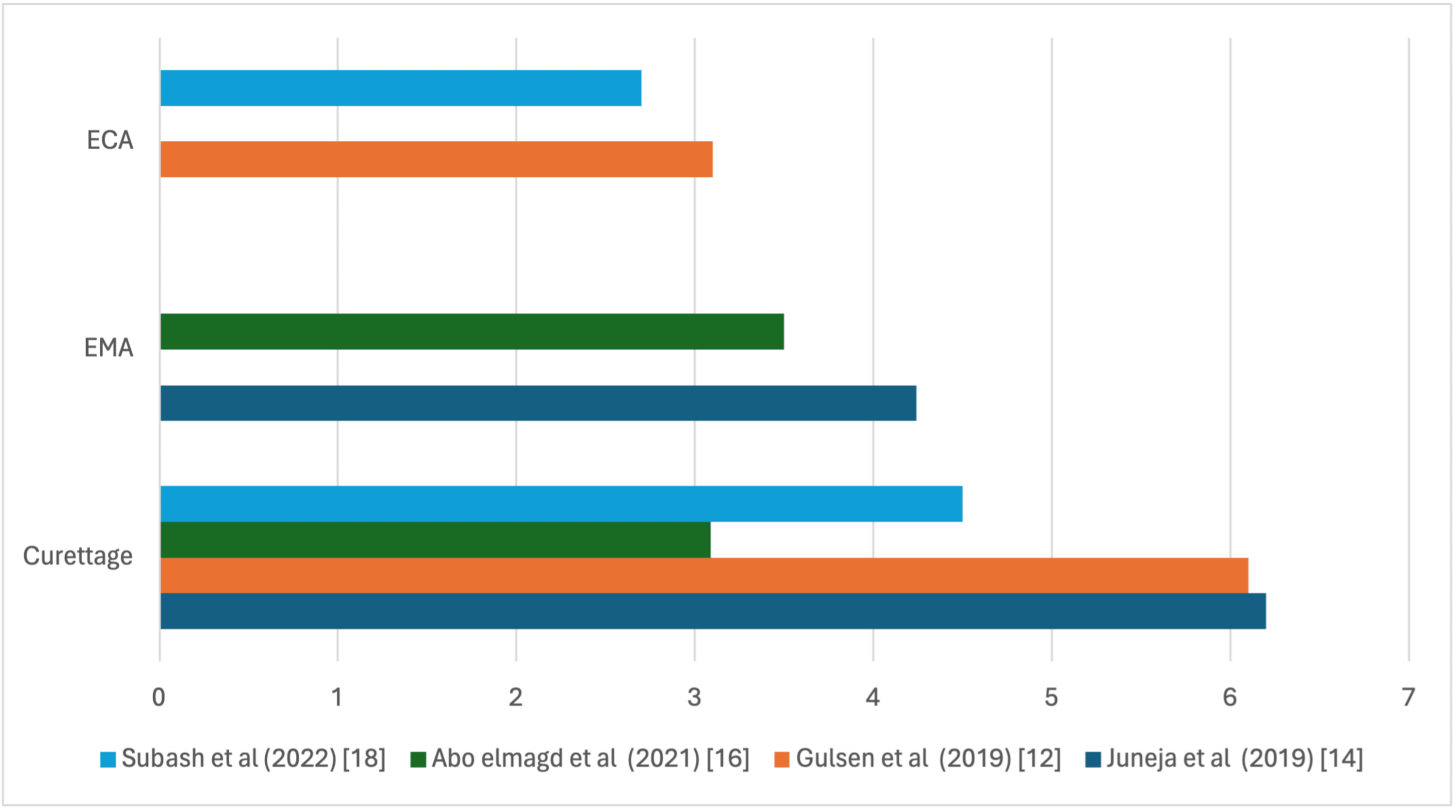
Evaluating mean pain score on postoperative day one using the numerical pain score (NPS). ECA: endoscopic coblation adenoidectomy, EMA: endoscopic microdebrider adenoidectomy

Singh et al. (2019) noted no statistical significance in recovery time between the two treatment modalities (p = 0.445) [15]. This contrasts with Abo Elmagd et al., who reported a mean post-op recovery of 2.8 days in the EMA group, in contrast to 8.23 days in the traditional curettage group (p<0.001) [16]. Although Abo Elmagd reported a remarkable difference in recovery time, there was no statistical difference in mean postoperative pain scores between the two treatment groups on POD1 and POD7 using NPS (P=0.65, P=0.79), respectively (Figure 4) [16]. 

**Figure 5:**
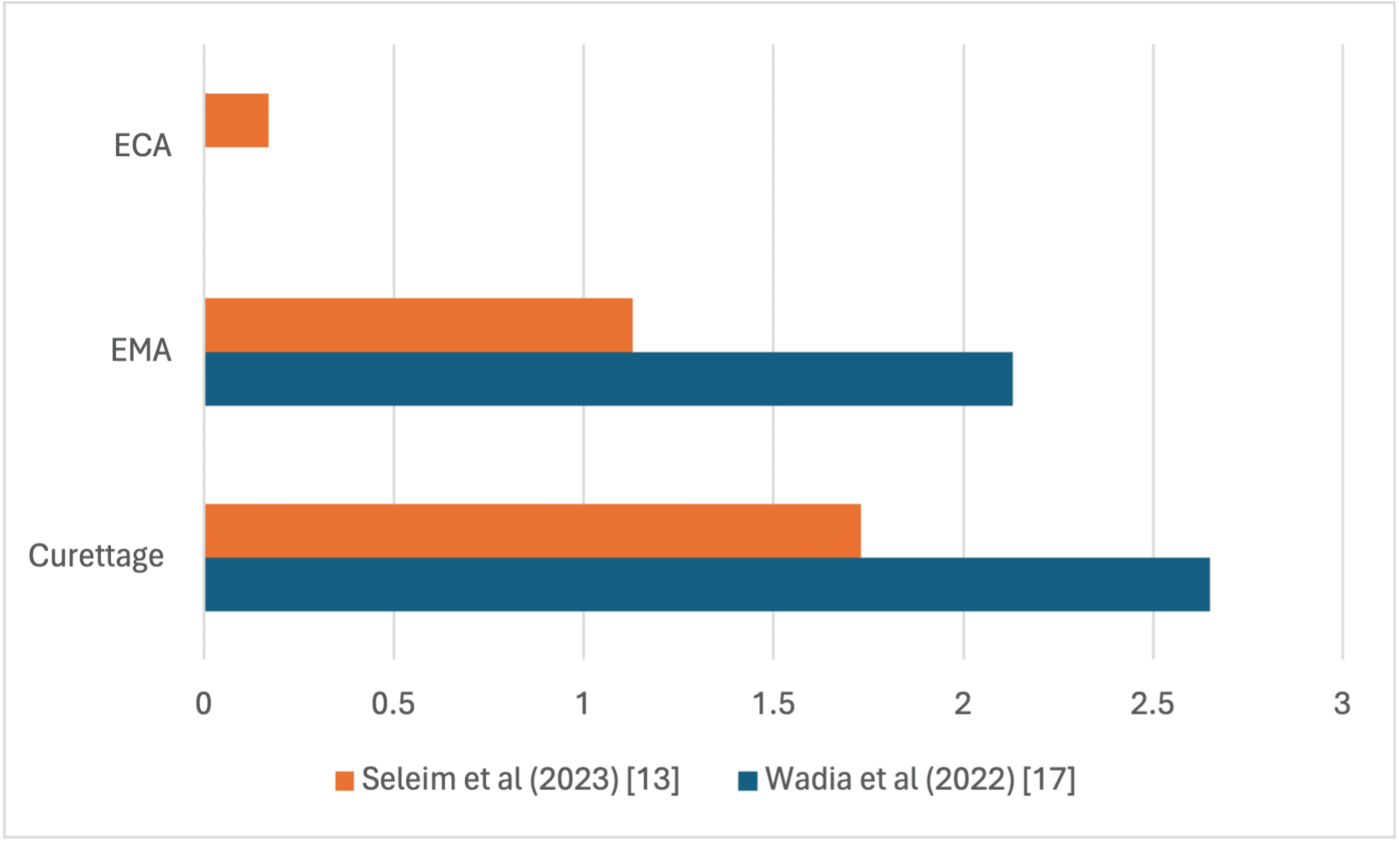
Evaluating mean pain score on postoperative day one using the visual assessment score (VAS). ECA: endoscopic coblation adenoidectomy, EMA: endoscopic microdebrider adenoidectomy

Wadia et al. used the VAS and reported no statistical difference between the two groups (p=0.39) (Figure 5) [17]. This is consistent with the findings of Kozcu et al., who interestingly reported no statistical difference in WBF scores on the first day, but better NPS scores were reported on POD1, POD2 and POD3 in the EMA group (p=0.042, p<0.001, p=0.002) respectively [10]. Better WBF scores were also reported in the EMA group on POD2 and POD3 (p=0.041, p=0.022). No difference was reported in NPS and WBF on day 4 to day 10 post-op. Juneja et al. reported similar findings as Kozcu et al., the EMA group had better pain scores compared to conventional curettage (p<0.05) [14].

ECA Versus Traditional Curettage 

Both Gulsen et al. and Subash et al. used NPS to compare post-operative pain between the ECA group and traditional curettage. The findings of the two RCTs are consistent, reporting lower pain scores in the ECA group. Both Gulsen et al. and Subash et al. reported significant difference on POD1 (p < 0.022, p<0.002) respectively [12,18]. However, there was no significant difference in pain after the first two post-operative days according to Gulsen et al.

EMA, ECA and Curettage

Seleim et al. used VAS to compare the three treatment modalities. ECA was found to have the best scores on POD1 and POD7 (p<0.001, p<0.003) respectively [13]. Singh et al. (2020) used WBFs to compare ECA to EMA, and the findings were consistent with the findings of Seleim et al., as during the first 24 and 72 hours post-op, ECA was superior to EMA (p<0.05) [13].

Residual Adenoid Tissue 

Eight papers reported residual adenoid tissue. Endoscopic techniques were found superior in resecting adenoid tissues in the eight papers that reported this surgical outcome. Five papers compared EMA to traditional curettage and a significant difference was reported. Juneja et al., Singh et al. (2019), Rajan et al. and Abo Elmagd et al. used a post-procedural endoscopy and reported complete or near-complete resection of adenoids in the EMA group (p=0.005, p=<0.001, p=0.01) respectively [11,14-16]. Wadia et al. reported that with EMA, complete resection was achieved in 83.3% of cases compared to 53.3% in the conventional group (p<0.05) [17]. 

Gulsen et al. compared curettage to ECA, and while 22.2% of the curettage group had residual adenoids, no patients in the ECA group had residual tissue left [12]. Singh et al. (2020) compared ECA to EMA and reported that 98.5 of adenoids were removed in the ECA group, compared to 95.7 in the EMA [19]. 

Discussion 

This systematic review compared postoperative outcomes across adenoidectomy techniques. Endoscopic approaches achieved more complete adenoid removal and lower early postoperative pain, albeit with longer operative times compared with conventional curettage.

Endoscopic techniques generally demonstrated superior postoperative middle ear pressure, likely reflecting reduced local trauma and edema. Improved visualization and more precise tissue removal during endoscopic procedures may help preserve Eustachian tube function [20]. These findings are further supported by Giri et al., who reported improved middle ear pressure in the EMA group at one month postoperatively, although the differences were no longer statistically significant at the two-month follow-up [21]. Variations in pre- and postoperative care - such as the use of lidocaine and adrenaline in the curettage group or oxymetazoline in the endoscopic groups [10,12] - may have influenced these outcomes. Seleim et al. similarly reported no significant differences among EMA, ECA, and curettage at two weeks and three months, although tympanogram graphs rather than exact pressure measurements were used, and natural normalization over time may have masked early differences [13]. 

Regarding hearing outcomes, Rajan et al. found no significant difference between EMA and traditional curettage at 12 weeks (P=0.27). In contrast, a prospective study reported better hearing in the EMA group at one month (p=0.027), with the difference maintained at two months (p=0.004) [21]. This discrepancy may reflect differences in follow-up duration, as transient hearing abnormalities may have resolved by 12 weeks postoperatively.

With respect to intraoperative time, endoscopic techniques, including EMA and ECA, consistently required longer operating times than conventional curettage. This likely reflects the additional time required for setup and handling of endoscopic equipment, as well as the more thorough adenoid removal achievable with these techniques [16].

Despite longer operative times, endoscopic approaches were associated with lower early postoperative pain (Figures 4, 5). Coblation techniques, in particular, demonstrated the lowest pain scores [12,13,18]. While these differences persisted on postoperative days 2 and 3, variability emerged in later measurements, with some studies reporting no significant differences after day 3 [12], and others showing benefits maintained up to day 7 [13]. Such variability may be influenced by differences in pain scoring systems, follow-up schedules, patient age, pain thresholds, and surgeon experience. Additionally, postoperative analgesia protocols were inconsistently reported, further contributing to variability. 

Endoscopic techniques consistently achieved near-complete adenoid resection compared with traditional curettage [11,12,14-17,19], likely due to improved visualization [16]. Complete resection is important to reduce the risk of persistent symptoms or adenoid regrowth.

It should be noted that considerable variability was observed among the included studies. Differences in surgical expertise, perioperative protocols, and methods of outcome assessment-particularly for pain and middle ear pressure-may have contributed to the heterogeneity of results. Variations in patient selection and follow-up duration may also explain the inconsistent findings across studies.

Recent reviews support these findings, indicating that coblation adenoidectomy provides more complete adenoid removal than traditional curettage, which may improve nasal obstruction and reduce revision rates [22,23]. Coblation may also confer modest advantages in intraoperative bleeding and early postoperative pain, although the clinical significance is limited, as adenoidectomy is generally not highly painful [22]. Overall, these observations suggest that endoscopic techniques achieve more complete resection while functional outcomes remain largely comparable to conventional curettage.

Limitations 

This systematic review has several notable limitations. Firstly, the included studies exhibited heterogeneity in terms of surgical techniques, outcome measures, and follow-up periods, which may affect the comparability of results. Additionally, most studies had relatively small sample sizes and variable methodological quality, potentially influencing the robustness of the conclusions. The inconsistency in the use of pain scoring systems and the lack of standardized postoperative care further introduce variability in outcome assessment.

A notable limitation is the geographic concentration of the included studies, with the majority originating from Turkey, India, and Egypt. This imbalance may limit the generalizability of the findings to other populations and could reflect regional differences in research output, clinical practice, and adoption of specific surgical techniques. Therefore, the applicability of these results to broader or more diverse pediatric populations should be interpreted with caution.

Moreover, there is limited data on the long-term effects and cost-effectiveness of endoscopic techniques, which are important factors for clinical decision-making. Finally, potential publication bias cannot be excluded, as studies with favorable results are more likely to be published. Future high-quality, large-scale randomized trials with standardized outcome measures are necessary to strengthen the evidence base.

## Conclusions

Adenoidectomy remains a routine pediatric procedure with evolving surgical techniques aimed at improving safety and recovery. Endoscopic approaches, particularly coblation, were associated with superior immediate postoperative outcomes, including reduced pain and improved middle ear pressure within the first few days after surgery. However, these advantages were largely transient and not statistically significant beyond the early postoperative period. Endoscopic coblation also demonstrated the most complete adenoid removal and the lowest intraoperative bleeding, though at the expense of longer operative time.

While functional outcomes such as middle ear pressure and hearing thresholds tend to normalize over time regardless of technique, endoscopic visualization provides a clear advantage in ensuring completeness of resection. Nonetheless, the clinical significance of early postoperative benefits remains limited, given their transient nature and the overall safety of all three methods.

Cost-effectiveness and long-term outcomes were inadequately addressed in the available literature, and geographic variability in study origin limits generalizability. Future large-scale, multicenter randomized controlled trials with standardized pain scoring systems and uniform follow-up durations are warranted to better define the optimal surgical technique for pediatric adenoidectomy.
